# Elevated circulating metalloproteinase 7 predicts recurrent cardiovascular events in patients with carotid stenosis: a prospective cohort study

**DOI:** 10.1186/s12872-020-01387-3

**Published:** 2020-02-26

**Authors:** David Moreno-Ajona, Pablo Irimia, José Antonio Rodríguez, María José García-Velloso, Jesús López-Fidalgo, Leopoldo Fernández-Alonso, Lukasz Grochowitz, Roberto Muñoz, Pablo Domínguez, Jaime Gállego-Culleré, Eduardo Martínez-Vila

**Affiliations:** 1 Department of Neurology, Clínica Universidad de Navarra, Av. Pío XII 36, 31008 Pamplona, Navarra Spain; 2 Instituto de Investigación Sanitaria de Navarra (IDISNA), Pamplona, Spain; 3 Red de Investigación Cooperativa de Enfermedades Vasculares Cerebrales (INVICTUS PLUS), Madrid, Spain; 4 Laboratory of Atherothrombosis, Program of Cardiovascular Diseases, CIMA-Universidad de Navarra, 31008 Pamplona, Spain; 5 CIBERCV, Madrid, Spain; 6 Department of Nuclear Medicine, Clínica Universidad de Navarra, 31008 Pamplona, Spain; 7 Universidad de Navarra, ICS, Unidad de Estadística, 31008 Pamplona, Spain; 8 Department of Vascular Surgery, Complejo Hospitalario de Navarra, 31008 Pamplona, Spain; 9 Department of Vascular Surgery, Clínica Universidad de Navarra, 31008 Pamplona, Spain; 10 Department of Neurology, Complejo Hospitalario de Navarra, 31008 Pamplona, Spain; 11 Department of Radiology, Clínica Universidad de Navarra, 31008 Pamplona, Spain

**Keywords:** Biomarker, Carotid endarterectomy, Matrix metalloproteinases, Positron emission tomography

## Abstract

**Background:**

Major adverse cardiovascular events are the main cause of morbidity and mortality over the long term in patients undergoing carotid endarterectomy. There are few reports assessing the prognostic value of markers of inflammation in relation to the risk of cardiovascular disease after carotid endarterectomy. Here, we aimed to determine whether matrix metalloproteinases (MMP-1, MMP-2, MMP-7, MMP-9 and MMP-10), tissue inhibitor of MMPs (TIMP-1) and in vivo inflammation studied by ^18^F-FDG-PET/CT predict recurrent cardiovascular events in patients with carotid stenosis who underwent endarterectomy.

**Methods:**

This prospective cohort study was carried out on 31 consecutive patients with symptomatic (23/31) or asymptomatic (8/31) severe (> 70%) carotid stenosis who were scheduled for carotid endarterectomy between July 2013 and March 2016. In addition, 26 healthy controls were included in the study. Plasma and serum samples were collected 2 days prior to surgery and tested for MMP-1, MMP-2, MMP-7, MMP-9, MMP-10, TIMP-1, high-density lipoprotein, low-density lipoprotein, high-sensitivity C-reactive protein and erythrocyte sedimentation rate. ^18^F-FDG-PET/CT focusing on several territories’ vascular wall metabolism was performed on 29 of the patients because of no presurgical availability in 2 symptomatic patients. Histological and immunohistochemical studies were performed with antibodies targeting MMP-10, MMP-9, TIMP-1 and CD68.

**Results:**

The patients with carotid stenosis had significantly more circulating MMP-1, MMP-7 and MMP-10 than the healthy controls. Intraplaque TIMP-1 was correlated with its plasma level (*r* = 0.42 *P* = .02) and with ^18^F-FDG uptake (*r* = 0.38 *P* = .05). We did not find any correlation between circulating MMPs and in vivo carotid plaque metabolism assessed by ^18^F-FDG-PET. After a median follow-up of 1077 days, 4 cerebrovascular, 7 cardiovascular and 11 peripheral vascular events requiring hospitalization were registered. Circulating MMP-7 was capable of predicting events over and above the traditional risk factors (HR = 1.15 *P* = .006). When the model was associated with the variables of interest, the risk predicted by ^18^F-FDG-PET was not significant.

**Conclusions:**

Circulating MMP-7 may represent a novel marker for recurrent cardiovascular events in patients with moderate to severe carotid stenosis. MMP-7 may reflect the atherosclerotic burden but not plaque inflammation in this specific vascular territory.

## Background

Atherosclerotic disease of the carotid artery is responsible for 30% of ischemic strokes [1]. Indeed, patients undergoing carotid endarterectomy have been shown to be at risk of major adverse cardiovascular events [2]. History of peripheral artery disease [2], chronic renal insufficiency [3] and ischaemic heart disease [4] have been identified as predictors of cardiovascular events following endarterectomy.

Inflammation and proteolytic activity are strongly associated with plaque instability and rupture and may predict the risk of cardiovascular events [5, 6]. However, there are few reports assessing the prognostic value of markers of inflammation in relation to the risk of cardiovascular disease after carotid endarterectomy [7].

Defects in the synthesis and breakdown of the extracellular matrix are considered key factors in the development of atherosclerotic disease and its thrombotic complications, and metalloproteinases (MMPs) are important mediators of these processes [8]. The relevance of MMPs in atherosclerosis has raised the possibility that they may be used as cardiovascular biomarkers [9]. Several studies have shown that different MMPs are more prominent in the blood of patients with carotid atherosclerosis or ischaemic stroke [7, 10–13]. Higher circulating levels of MMP-2, MMP-9, MMP-7, MMP-12, and TIMP-1 (tissue inhibitor of matrix metalloproteinase 1) might contribute to carotid plaque instability due to inflammatory mechanisms [7, 10]. However, studies into the relationship between MMPs and the metabolic activity of the vascular wall, measured by ^18^fluorine-fluorodeoxyglucose-positron emission tomography combined with computed tomography (^18^F-FDG-PET/CT), as an indirect measure of carotid inflammation in vivo, have produced conflicting results [11, 12, 14].

Here, we aimed to determine whether matrix metalloproteinases (MMP-1, MMP-2, MMP-7, MMP-9 and MMP-10), tissue inhibitor of MMPs (TIMP-1) and vascular ^18^F-FDG-PET/CT predict recurrent cardiovascular events in patients with carotid stenosis who underwent endarterectomy.

## Methods

### Study population

In this prospective, longitudinal, hospital-based cohort study, 31 consecutive patients with symptomatic or asymptomatic severe (> 70%) carotid stenosis who were scheduled for carotid endarterectomy at Clínica Universidad de Navarra or Hospital de Navarra (Spain) were recruited between July 2013 and March 2016. No patient was included postoperatively, and patients were excluded if there was evidence of active infection, generalized or local inflammatory disease (discounting atherosclerosis), chronic kidney disease, or neoplastic disease or if they were on haemodialysis. A complete medical history was recorded for all patients, including details of previous stroke, transient ischaemic attack, coronary disease (myocardial infarction, angina or revascularization), intermittent claudication, arterial hypertension, smoking status, diabetes mellitus, body mass index, and grade of bilateral carotid stenosis and medication. Patients were selected for endarterectomy based on consensus among the multidisciplinary team members of the Carotid Clinic, which included vascular surgeons, cardiologists, and neurologists, who evaluated the patients before surgery. All procedures were performed under general anaesthesia by the vascular surgeons who have extensive experience in carotid endarterectomy. A blood analysis was performed no more than 2 days prior to surgery. Active smokers or patients who had stopped smoking within 2 years of recruitment were considered smokers. A carotid ultrasonography assessment was performed to determine the grade of stenosis, according to the latest guidelines [15], and this was confirmed by magnetic resonance angiography with coronal fast 3D T1-weighted sequences after administration of a single dose of intravenous gadolinium contrast, with digital subtraction and maximum-intensity projection reconstructions, using a 3-T MRI scanner (Magnetom Trio Tim, Siemens, Erlanger, Germany). Stenosis was measured following NASCET recommendations [16]. The patients were classified as symptomatic if they had had a stroke, transient ischemic attack (TIA) or *amaurosis fugax* ipsilateral to the stenotic carotid artery. Carotid ultrasonography in these patients was performed as part of routine clinical practice in the stroke unit. Asymptomatic carotid stenosis was detected in patients with a high cardiovascular risk undergoing carotid ultrasonography for screening. History of previous stroke in asymptomatic patients included non-atherosclerotic stroke. One “asymptomatic patient” was diagnosed with cancer following ^18^F-FDG-PET/CT imaging at the beginning of the study and was therefore excluded from the cohort. In addition, from a cohort of healthy individuals free from cardiovascular diseases, workers at the University of Navarra (Spain) who attended the Internal Medicine outpatient clinic for a general check-up, 26 subjects age and sex-matched were included (mean age 66.8 ± 3.0, males/females 16/10) for measurement of blood MMP-1, MMP-2, MMP-7, MMP-9, MMP-10 and TIMP-1. All protocols were approved by the Committee for Medical and Research Ethics. Participants gave written informed consent. Samples from patients included in the study were provided by the Biobank of the University of Navarra and were processed following standard operating procedures approved by the Research Ethics and Scientific Committees.

### Biomarker assessment

Blood samples were collected after overnight fasting, no more than 2 days prior to carotid endarterectomy. The blood was allowed to clot and was centrifuged at 2000 x *g* for 10 min using a refrigerated centrifuge. The serum was removed, aliquoted and stored at − 80 °C until it was assayed. Plasma was obtained from citrated blood by centrifugation at 2000 x *g* for 10 min using a refrigerated centrifuge, aliquoted and stored at − 80 °C until assayed. MMP-1, MMP-2, MMP-7, MMP-9 and MMP-10 were measured in citrated plasma with a bead-based multiplex assay using Luminex technology (MILLIPLEX MAP Human MMP Magnetic Bead Panel 2, ref. HMMP2MAG-55 K; Merck, Darmstadt, Germany). TIMP-1 was assayed in serum using a specific enzyme-linked immunosorbent assay (Human TIMP-1 Quantikine ELISA Kit, ref. DTM100; R&D Systems, Minneapolis, USA). MMPs and TIMP-1 were measured following the manufacturers’ instructions. The inter- and intra-assay coefficients of variation for these ELISAs were < 6%.

Total serum cholesterol, high-density lipoprotein (HDL) cholesterol and triglycerides were measured in fasting blood samples using standard laboratory techniques. Low-density lipoprotein (LDL) cholesterol was estimated using the Friedewald equation, and high-sensitivity C-reactive protein (hs-CRP) was measured in an immunoassay (Immulite; Diagnostic Products Corporation, Los Angeles, CA, USA). The erythrocyte sedimentation rate (ESR) was also analysed by the Westergren method [17].

### Histological assessment

Vascular segments were removed carefully at endarterectomy to preserve plaque structure. Specimens from the 31 patients undergoing carotid endarterectomy were cut into transverse pieces and fixed immediately in 4% paraformaldehyde, decalcified for 24 to 72 h at room temperature (Osteosoft: Merck Millipore, Darmstadt, Germany) and embedded in paraffin. Haematoxylin-eosin and van Gieson staining were used to examine the microscopic morphological features of the tissue (Nikon Optiphot-2, Japan).

For protein immunolocalization, serial tissue sections from all patients were analysed by immunohistochemistry. Antigen retrieval was performed using 10 mM citrate, pH 6.0, (MMP-10) or TE, pH 9.0, (MMP-9, TIMP-1, CD68) and a DakoCytomation Pascal pressure chamber (Dako) at 95 °C for 30 min. Slides were treated with H_2_O_2_ for 10 min to block endogenous peroxidase activity, and sections were blocked with 5% normal goat serum in PBS. Tissue sections were incubated with antibodies targeting MMP-10 (rabbit polyclonal, Acris ref. AP07210PU-N, dil. 1:20), MMP-9 (rabbit polyclonal, Thermo Fisher ref. PA5–13199, dil. 1:800), TIMP-1 (mouse monoclonal, Merck ref. MAB13429, dil. 1:400) and CD68 (mouse monoclonal, Dako ref. GA609, dil. 1:2000), as described previously [18]. After washing, sections were incubated for 30 min with HRP-labelled polymer conjugated to anti-rabbit (ref. K4003) or anti-mouse (ref. 4001) secondary antibodies (Dako). The signal was revealed by using the DAB+ Substrate Chromogen System (Dako ref. K3468). Negative controls, prepared by omitting the primary antibody, were included in each processed batch. Three independent observers, blinded to clinical data, used a semi-quantitative visual grading scale (Additional file 2) to assess the immunostaining (0–5 scale).

### Vascular ^18^F-FDG PET/CT imaging

**Fig. 1:**
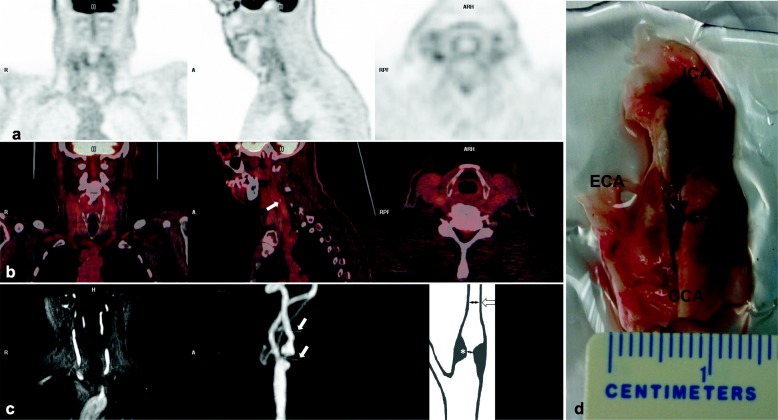
Carotid imaging prior to endarterectomy was assessed by 18-FDG-PET combined with (**a**, **b**). ^18^F-FDG-PET combined with CT enables the image fusion of both techniques and localizes calcium (white arrow) (**b**). The degree of stenosis was confirmed by magnetic resonance angiography with raw images and maximum intensity projection with measurement of the stenosis (arrows). A schematic drawing of the measurement of the stenosis is shown (**c**). Stenosis was measured following NASCET recommendations, where the point of maximum stenosis (*) is compared with the distal lumen of the ICA (arrow). Atheroma plaque removed by endarterectomy; the surgery was performed within 1 week of imaging (**d**). ICA: internal carotid artery; ECA: external carotid artery; CCA: common carotid artery

^18^F-FDG-PET/CT imaging that focused on carotid wall metabolism was performed in 29 of the 31 patients (Fig. 1), as there was no presurgical availability in 2 symptomatic patients. The maximum standardized uptake value (SUVmax) was used to measure the ^18^F-FDG uptake at the ipsilateral and contralateral carotid arteries. The target-to-background ratio (TBR) was calculated as the ratio of SUVmax measured in the atherosclerotic plaque or the arterial vessel to the venous blood pool SUVmax to correct for blood-pool uptake. Venous activity was measured as mean SUV in the ipsilateral jugular veins for carotid studies, and inferior vena cava for the other territories (aortic arch and the abdominal aorta).

### Patient follow-up

Patients were followed at the outpatient service of the Department of Neurology and/or Vascular Surgery every 3 or 6 months and at the Emergency Department (median follow-up 1077 days [interquartile range, 689–1484 days]) until May 2018. The primary outcome of the study was defined as the composite of cardiovascular death, a nonfatal cardiovascular event (including stroke and myocardial infarction) or rehospitalization for acute ischaemia (TIA, *amaurosis fugax, angina pectoris* and peripheral artery disease) as previously described [19]. These outcomes were determined through a review of the patient’s medical history. Peripheral artery disease was diagnosed with the combination of compatible symptoms, physical examination, ankle brachial index and duplex ultrasound [20]. Additional imaging (with CT angiography, MR angiography, or digital subtraction angiography) was performed when revascularization was considered [21]. Patients with symptoms of coronary disease were reviewed by the cardiology team, and coronariography or other complementary tests were performed in accordance with the latest guidelines [22]. The period between surgery and the day of outcome was calculated, and the overall survival and presence of recurrent cardiovascular events were calculated from the day of surgery to the date of the last follow-up available. No patients were lost to follow-up.

### Statistical methods

All categorical variables are expressed as frequencies (ratios), and all the continuous variables are expressed as the mean (± standard error). Statistical analysis included a Shapiro-Wilk test for normality. Pearson’s correlation coefficient and Spearman’s correlation coefficient were used to test the correlations between MMPs and SUVmax or TBR. A Bonferroni correction for multiple comparisons (using as a reference for the *p*-value 0.05 divided by the number of comparisons) was made. An unpaired Student’s t test was used to compare the MMP values between the patients (symptomatic and asymptomatic combined) and healthy controls, and a Cox regression for recurrent events with multiple imputation for missing data was used to determine the role of MMPs in event prediction. Hazard ratios were obtained after adjusting for relevant covariates. Receiver operating characteristic (ROC) curves were used to analyse the ability of MMP-7 to predict events in the population of subjects after fitting a logistic regression model. Risk factors, including age, sex, tobacco use, cholesterol levels, hypertension and diabetes mellitus, were grouped following the recommendations of a previous study [23]. Considering that the population of Navarra has higher levels of HDL-cholesterol on average, a specifically adapted version of the Framingham-Wilson Coronary Risk Equation was used to assess global cardiovascular risk: RIsk score for CORonary events in the population of NAvarra (RICORNA) [24].

Statistical significance was established at *p* < .05, and the statistical analysis, including sample size computations, was performed with the Stata software package version 15.1 for Mac (StataCorp; College Station, TX, USA).

The size of the available sample is rather small, which was the reason some non-parametric analysis was performed. For a Cox regression analysis, this sample size jointly with a significance level of 0.05 and a test power of 0.20 allows the detection of hazard ratios of size less than 0.0001 assuming a standard deviation of 0.04, which is quite good since for that we have obtained a hazard ratio of 0.006. For the worst standard deviation (we have 2.7), a hazard ratio of size 0.8300 could be detected, which is reasonably good since we obtained a hazard ratio of 1.7.

## Results

This study cohort was composed of 31 subjects (mean age ± standard deviation 67.4 ± 2.6 years, 21/31 men) after the exclusion of one patient due to the diagnosis of bladder cancer following the ^18^F-FDG-PET/CT study. A similar population of 26 healthy controls (mean age 66.8 ± 3.0 years, 16/26 men) was also analysed. Among the patients, 23/31 were symptomatic (see “Study population” above), and 8/31 were asymptomatic (see Table 1 for the patients’ characteristics). The rates of hypertension and diabetes mellitus were similar, whereas other risk factors differed between the two groups (Table 1). When the baseline plasma levels of MMPs and TIMP-1 were compared, significantly higher levels of MMP-1, MMP-7 and MMP-10 were evident in the patient group, while no such differences were found for MMP-2, MMP-9 or TIMP-1 (Table 2).

**Table 1:** Patients’ characteristics

Clinical history and characteristics	Symptomatic (*n* = 23)	Asymptomatic (*n* = 8)		Controls (*n* = 26)	
Age, years (SD)	67.3 (16.0)	66.3 (22.5)	*p =* .4	66.8 (3.0)	*P =* .4
Male (%)	15 (65)	6 (75)	*p =* .3	16 (62)	*P =* .3
Hypertension (%)	17 (74)	6 (75)	*p =* .6	0	
Diabetes mellitus (%)	8 (35)	2 (10)	*p =* .6	0	
Prediabetes (%)	1 (4)	2 (25)	*p =* .12	0	
Coronary disease (%)	7 (30)	1 (12)	*p =* .4	0	
Tobacco (%)	12 (52)	7 (87)	*p =* .1	0	
Previous stroke/TIA (%)	4 (17)	2 (25)	*p =* .5	0	
BMI, kg/m^2^ (SD)	27.5 (1.1)	29.2 (1.3)	*p =* .7	N/A	
Grade of ipsilateral stenosis % (SD)	82.6 (16.2)	85.0 (14.2)	*p =* .6	N/A	
Grade of contralateral stenosis % (SD)	37.4 (25.0)	35.0 (23.2)	*p =* .7	N/A	
Medication					
Statins (%)	13 (57)	7 (87)	*p =* .4	0	
Anti-aggregants (%)	11 (48)	7 (87)	***p =*** **.04**	0	
Anti-coagulants (%)	0	1 (12)	***p =*** **.02**	0	
Blood analysis					
Leukocytes, 10^9^/l (SD)	7.69 (0.43)	7.50 (0.73)	*p =* .4	N/A	
Platelets, 10^9^/l (SD)	210.3 (10.1)	210.7 (13.5)	*p =* .5	N/A	
ESR, mm/h (SD)	10.86 (1.30)	4.83 (1.25)	***p =*** **.03**	N/A	
hs-CRP, mg/l (SD)	1.94 (0.41)	1.38 (0.74)	*p =* .3	N/A	
LDL cholesterol, mg/dl (SD)	102.3 (6.2)	80.0 (10.0)	***p =*** **.04**	N/A	
HDL cholesterol, mg/dl (SD)	44.3 (2.4)	49.7 (5.8)	*p =* .8	N/A	
Triglycerides, mg/dl (SD)	119.4 (14.5)	134.6 (27.4)	*p =* .6	N/A	

*SD* standard deviation, *TIA* transient ischemic attack, *BMI* body mass index, *N/A* not applicable, *ESR* erythrocyte sedimentation rate, *hs-CRP* high-sensitivity C-reactive protein, *LDL* low-density lipoprotein, *HDL* high-density lipoprotein, *unk* unknown, *N/A*: not applicable. Data are expressed as the total number (percentage) or mean (standard deviation)

**Table 2:** Baseline plasma levels of MMPs and TIMP-1

Biomarker	Patients (*n* = 31)	Controls (*n* = 26)	
MMP-1, ng/ml	11.8 (9.5)	6.2 (4.1)	***p*** **= .003**
MMP-2, ng/ml	125.3 (6.5)	135 (6.4)	*p* = .3
MMP-7, ng/ml	20.4 (6.4)	17.5 (4.1)	***p*** **= .02**
MMP-9, ng/ml	117.8 (10.7)	123.5 (12.4)	*p* = .7
MMP-10, pg/ml	726.7 (81.6)	513.2 (40.0)	***p*** **= .01**
TIMP-1, ng/ml	211.6 (9.4)	195.5 (8.1)	*p* = .2

Data are expressed as the mean (standard deviation). *MMP* metalloproteinase, *TIMP* tissue inhibitor of matrix metalloproteinases

### MMPs and ^18^F-FDG PET/CT imaging

No significant correlation was found between blood levels of MMPs and the SUV of the symptomatic carotid artery (for MMP-7, *r* = − 0.20 *p* = .28) or the contralateral carotid artery (for MMP-7, *r* = − 0.23 *p* = .21). Similar results were obtained with the normalized TBR for the ipsilateral carotid artery (for MMP-7, *r* = − 0.35 *p* = .06) or for the contralateral carotid artery (for MMP-7, *r* = − 0.24 *p* = .21). SUVmax values were consistently correlated between neighbouring territories (carotid and aortic arch, *r* = 0.41 *p* = .03; carotid and abdominal aorta, *r* = 0.51 *p* = .02). However, none of the blood levels of MMPs was correlated with ^18^F-FDG uptake in the carotid territory, aortic arch or abdominal aorta (see table IIS in the supplementary material).

### MMPs and histological findings

All endarterectomy samples corresponded to advanced atherosclerotic plaques (types V–VIII) according to the American Heart Association (AHA) histological classification [25]. The median of the semi-quantitative visual grading scale (0–5) for each MMP immunostaining is available in Table IS in the supplementary material. There was a significant association between CD68 and MMP-9 (r_S_ = 0.42, *p* = 0.009) or TIMP-1 (r_S_ = 0.38, *p* = .018), even after Bonferroni correction, but not with MMP-10 (r_S_ = 0.28). A moderate correlation was found between plasma and intraplaque TIMP-1 values (*r* = 0.42; *p* = .02), but there was no association between intraplaque MMP-9 or MMP-10 and their plasma concentration. The correlations between plasma MMPs and histological findings are shown in Table 3. Intraplaque TIMP-1 was correlated with its plasma level (*r* = 0.42 *p* = .02) and with ^18^F-FDG uptake (*r* = 0.38 *p* = .05). A weak correlation did appear to exist between intraplaque CD68 (macrophage marker) and ^18^F-FDG-PET (*r* = 0.31), yet this was not statistically significant (*p* = .12). No correlation was found between intraplaque MMPs, TIMP-1, CD68 and hs-CRP or ESR.

**Table 3:** Correlation between plasma MMPs and histological findings

Histology	Plasma	Correlation	*P*-value
CD68	MMP-9	r_S=_0.42	**0.009**
	MMP-10	r_S=_0.28	0.09
	MMP-7	r_S=_0.04	0.83
	TIMP-1	r_S=_0.38	**0.018**
MMP-10	MMP-10	r_S=_0.14	0.45
MMP-9	MMP-9	*r*_=_0.37	0.08
TIMP-1	TIMP-1	*r* = 0.42	**0.02**

### MMPs as predictors of recurrent cardiovascular events

**Fig. 2:**
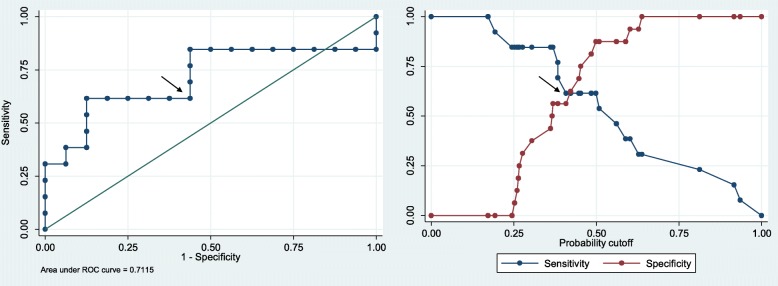
The arrows indicate the cut-off point in both graphs, chosen as the maximum of the ROC curve (left) with respect to the diagonal. The graph on the right reflects the sensitivity and specificity: the cut-off point has both good sensitivity and specificity compared with the intersection point

After a mean follow-up of 1004 days, 4 cerebrovascular (2 ischemic strokes and 2 transient ischaemic attacks), 7 cardiovascular (4 myocardial infarctions, 3 *angina pectoris*) and 11 peripheral vascular events requiring hospitalization were registered. No deaths were recorded during the follow-up. As expected, the RICORNA risk score predicted future events (HR = 1.08 *p =* .002). When other variables were added to the model, including MMPs and the results of ^18^F-FDG-PET, circulating MMP-7 was the only MMP capable of improving the predictive value of RICORNA (HR = 1.15 *p* = .006). The TBR of the contralateral carotid artery alone seemed to be a good predictor of such events (HR = 3.5 *p* = .05), although when the model was associated with the variables of interest, namely, RICORNA and MMP-7, the risk was not significant for TBR (HR = 2.5 *p* = .15). The inclusion of other variables, such as the remaining MMPs, TIMP-1, CRP or ESR, and ^18^F-FDG-PET, failed to improve the prediction of cardiovascular events. Twenty multiple imputation for the 8 missing values in the variable MMP-7 and ^18^F-FDG-PET were applied. When using only RICORNA, the model was predictive (*P* = 0.001) and when using MMP-7 and ^18^F-FDG-PET the model was also predictive (*P* = 0.019). This proves the good predictive behaviour of MMP-7 thanks to the use of multiple imputation for missing data. MMP-7 levels were not significantly different in patients with and without coronary disease. When ROC curves were studied with a logistic regression for MMP-7, a cut-off point of 20.48 ng/ml was established (for a probability of .4986), which led to a sensitivity of 61%, a specificity of 87%, and a positive predictive value of 80%. This probability came from maximizing the ROC (Fig. 2).

## Discussion

In our cohort of patients, the circulating MMP-7 level, but not vascular ^18^F-FDG-PET/CT focused on the carotid wall, proved to be capable of predicting cardiovascular events after a 1004-day follow-up, improving the performance of the traditional cardiovascular risk factors. This novel finding is consistent with data from a previous study [7] in which high plasma MMP-7 levels were independently associated with total mortality after a 3.5-year mean follow-up. In contrast with our study, the authors did not document an association with cardiovascular events [7]. In a previous study involving 106 endarterectomies, overexpression of the MMP-7 gene was shown to be 100-fold higher in plaques than in normal arteries [26].

The mechanisms underlying the association between MMP-7 and recurrent cardiovascular events have yet to be defined. One possible explanation is that MMP-7 may increase plaque vulnerability through inflammatory processes [8, 11], yet we did not find any correlation between circulating MMP-7 and in vivo carotid plaque inflammation assessed by ^18^F-FDG-PET/CT. In low-inflammatory stages, ^18^F-FDG PET/CT may be less sensitive than histology; however, we did not find a correlation between circulating and intraplaque MMPs. We could not measure intraplaque MMP-7 and thereby correlate this to its plasma level to show a direct relationship with carotid disease. The absence of a correlation between MMP levels and ^18^F-FDG uptake in the carotid arteries, aortic arch or abdominal aorta suggests that high plasma level of MMP-7 may reflect the atherosclerosis burden but does not inform about the presence of plaque inflammation in a specific vascular territory. Additionally, arterial uptake of FDG is influenced by plaque composition. In line with our findings, the largest study to date evaluating the presence of vascular inflammation by ^18^F-FDG-PET/MRI in individuals with atheromatosis, showed that most FDG uptakes were detected in plaque-free arterial segments (suggesting an arterial inflammatory state at early stages of atherosclerosis) and in lipid-rich plaques [27]. Interestingly, previous studies performed in subjects with atherosclerotic disease did not observe a relationship between markers of systemic inflammation and 18F-FDG PET/CT in patients with carotid stenosis [11]. In the present study, all the samples corresponded to advanced calcified atherosclerotic plaques, in which an increase in calcium rarely overlaps with ^18^F-FDG uptake [12]. A recent international multicentre study showed that ^18^F-FDG-PET was capable of predicting stroke recurrence although this study had a higher incidence of cerebrovascular events [28] compared to our study.

MMPs are not only produced in conjunction with inflammation. An in vitro study found that enriching smooth muscle cell cultures with collagen VIII stimulated MMP production, which induced cell migration [29]. Furthermore, the actions of MMPs are not only local. In fact, MMP-7 is also related to the development of hyperlipidaemia and atherosclerosis through its cleavage of lipid-free apolipoprotein A-IV, an event that plays an important role in lipid metabolism and oxidant activity [30]. All things considered, the presence of other inflammatory cell types cannot be excluded, as histological analysis detected CD68, which is expressed by phagocytes.

The presence of higher circulating levels of MMP-1 and MMP-10 in patients with carotid stenosis compared to healthy controls is in line with previous studies [31, 32], highlighting the relationship between atherosclerosis and MMPs. Surprisingly, circulating TIMP-1, believed to inhibit MMP action and contribute to plaque stability, had a weak to moderate correlation with ^18^F-FDG-PET, probably due to the advanced stage of atherosclerosis.

The high rate of cardiovascular events we found can be explained by the extremely high prevalence of risk factors in our cohort and the systemic involvement of atherosclerosis. The presence of severe carotid stenosis has been proposed as a risk factor for the development of stroke, white matter disease [33], coronary artery disease [34], peripheral artery disease and all-cause mortality [35]. Atherosclerosis is a systemic vascular disorder potentially involving multiple vascular territories. Our data suggest that MMP-7 may be an important mediator in the relationship between carotid stenosis and the development of recurrent cardiovascular events in different vascular territories. Nevertheless, the utility of MMP-7 as a potential biomarker may also be related to its role in other medical conditions, such as heart failure [36] as well as oncological diseases [37] .

To date, several markers have been tested as potential tools to assess the risk of recurrent ischaemic stroke. The largest study tested 14 different biomarkers [38], including interleukin 6 and CRP [39], and limited utility in stroke prediction was found. Osteopontin, neopterin and myeloperoxidase [40], as well as lipoprotein-associated phospholipase A2 [41], free fatty acid levels [42] and circulating endothelial progenitor cells [43], have all been tested as recurrent stroke predictors with positive results, whereas the data regarding soluble CD40 ligand are inconsistent [40, 44]. Similar to MMP-7 in the present study, elevated lipoprotein (a) in patients with acute first ischaemic stroke [45] has been associated with a higher risk of combined recurrent cardiovascular events. However, none of these markers has been compared to MMP-7 in a similar setting, and therefore, larger studies including such patients should be performed.

The main strengths of this study are the detailed characterization of carotid plaque inflammation in vivo and the prolonged follow-up, with no loss. The main limitation of the study is the small number of patients, as larger studies may have identified differences in MMP-7 between symptomatic and asymptomatic patients or the utility of the other potential biomarkers. Indeed, the consequent lack of statistical power may have hindered the discovery of the potential predictive value of ^18^F-FDG-PET in cardiovascular events. In the histological study, the extensive decalcification required for the analysis of the endarterectomy samples may adversely affect tissue antigenicity, particularly that of extracellular proteins such as MMPs. Finally, peripheral artery disease is common in patients with carotid stenosis and we have not studied the presence of subclinical peripheral artery disease using ankle-brachial index, ultrasound, CT or MRI before endarterectomy [46]. Although our patients did not have symptoms of intermittent claudication, the presence of subclinical peripheral artery disease before endarterectomy cannot be completely excluded.

## Conclusions

Our findings provide evidence that circulating (plasma) MMP-7 could represent a novel marker for recurrent cardiovascular events in patients with severe carotid stenosis. While additional studies will determine the value of MMP-7 in clinical practice, our results support the potential utility of circulating MMP-7 as a risk biomarker over and above that of conventional cardiovascular risk factors. To validate and confirm these results, larger studies with more statistical power should be carried out.

## Supplementary information

**Additional file 1: Table IS.** Semiquantitative visual grading scale to assess immunostaining (0–5 scale). **Table IIS.** Correlations between blood levels of MMPs and ^18^F-FDG uptake.

**Additional file 2: Figures 1S, 2S, 3S** and **4S.** Representative images corresponding to the semiquantitative visual grading scale used to assess the immunostaining for CD-68, TIMP-1, MMP-9 and MMP-10.

## Data Availability

All data generated or analysed during this study are included in this published article.

## Abbreviations

^18^F-FDG PET/CT: ^18^Fluorine-fluorodeoxyglucose-positron emission tomography combined with computed tomography
AHA: American Heart Association
ESR: Erythrocyte sedimentation rate
HDL: High-density lipoprotein
hs-CRP: High-sensitivity C-reactive protein
LDL: Low-density lipoprotein cholesterol
MMPs: Metalloproteinases
NASCET: North American Symptomatic Carotid Endarterectomy Trial
RICORNA: Risk score for coronary events in the population of Navarra
ROC: Receiver operating characteristic
SUVmax: Standardized uptake value
TBR: Target-to-background ratio
TIA: Transient ischaemic attack
TIMP-1: Tissue inhibitor of matrix metalloproteinase 1
