# Supplementary material for: Elevated circulating metalloproteinase 7 predicts recurrent cardiovascular events in patients with carotid stenosis: a prospective cohort study
Source: BMC Cardiovasc Disord. 2020 Feb 26;20:93. doi: 10.1186/s12872-020-01387-3 (PMC7045396; doi:10.1186/s12872-020-01387-3)
Supplement: Supplementary file 1 — Additional file 1: Table IS. Semiquantitative visual grading scale to assess immunostaining (0–5 scale). Table IIS. Correlations between blood levels of MMPs and 18F-FDG uptake. [file 12872_2020_1387_MOESM1_ESM.docx]

**Table IS. Semiquantitative visual grading scale to assess immunostaining (0-5 scale).**

| **Patient** | **MMP-9** | **MMP-10** | **CD68** | **TIMP-1** |
| --- | --- | --- | --- | --- |
| **1** | 4.5 | 4.0 | 4.0 | 1.0 |
| **2** | 2.5 | 1.0 | 0.0 | 0.0 |
| **4** | 3.0 | 1.0 | 4.0 | 4.0 |
| **5** | 2.5 | 3.5 | 3.0 | 1.0 |
| **6** | 3.0 | 2.0 | 3.0 | 1.0 |
| **7** | 5.0 | 4.5 | 3.0 | 0.0 |
| **8** | 4.5 | 2.5 | 5.0 | 2.0 |
| **9** | 0.0 | 0.0 | 0 | 0.0 |
| **10** | 4.5 | 0.5 | 4.0 | 2.0 |
| **11** | 5.0 | 3.5 | 2.0 | 5.0 |
| **12** | 0.5 | 2.0 | 0.0 | 0.0 |
| **13** | 2.5 | 3.0 | 3.0 | 4.0 |
| **14** | 2.5 | 1.5 | 2.0 | 3.0 |
| **15** | 4.0 | 3.5 | 2.0 | 3.0 |
| **16** | 4.0 | 2.5 | 2.0 | 2.0 |
| **17** | 1.5 | 2.0 | 1.0 | 1.0 |
| **18** | 1.5 | 2.0 | 1.0 | 0,0 |
| **19** | 1,0 | 1.5 | 0.0 | 1.0 |
| **20** | 1.5 | 1.5 | 1.0 | 0,0 |
| **21** | 3.0 | 2.5 | 3.0 | 5.0 |
| **22** | 4.0 | 2.5 | 4.0 | 5.0 |
| **23** | 3.5 | 5.0 | 5.0 | 1.0 |
| **24** | 2.5 | 2.5 | 3.0 | 2.0 |
| **25** | 2.0 | 2.5 | 2.0 | 1.0 |
| **26** | 1.0 | 0.0 | 1.0 | 1.0 |
| **27** | 3.0 | 3.0 | 3.0 | 1.0 |
| **28** | 5.0 | 3.0 | 4.0 | 3.0 |
| **29** | 4.0 | 3.0 | 3.0 | 2.0 |
| **30** | 5.0 | 4.0 | 5.0 | 3.0 |
| **31** | 2.0 | 1.0 | 2.0 | 3.0 |

Table IIS. Correlations between blood levels of MMPs and ^18^F-FDG uptake.

|  | **Symptomatic carotid artery uptake** | **P-value** | **Aortic arch uptake** | **P-value** | **Abdominal aorta uptake** | **P-value** |
| --- | --- | --- | --- | --- | --- | --- |
| **MMP-1** | -0.05 | 0.77 | -0.07 | 0.68 | 0.15 | 0.4 |
| **MMP-2** | -0.01 | 0.95 | 0.01 | 0.95 | 0.05 | 0.78 |
| **MMP-7** | -0.20 | 0.28 | -0.28 | 0.12 | 0.12 | 0.53 |
| **MMP-9** | -0.28 | 0.13 | -0.11 | 0.57 | 0.09 | 0.62 |
| **MMP-10** | -0.19 | 0.33 | -0.14 | 0.46 | 0.07 | 0.71 |
| **TIMP-1** | 0.05 | 0.78 | -0.29 | 0.12 | -0.17 | 0.36 |
