# Supplementary material for: Elevated circulating metalloproteinase 7 predicts recurrent cardiovascular events in patients with carotid stenosis: a prospective cohort study
Source: BMC Cardiovasc Disord. 2020 Feb 26;20:93. doi: 10.1186/s12872-020-01387-3 (PMC7045396; doi:10.1186/s12872-020-01387-3)
Supplement: Supplementary file 2 — Additional file 2: Figures 1S, 2S, 3S and 4S. Representative images corresponding to the semiquantitative visual grading scale used to assess the immunostaining for CD-68, TIMP-1, MMP-9 and MMP-10. [file 12872_2020_1387_MOESM2_ESM.pptx]

## Slide 1
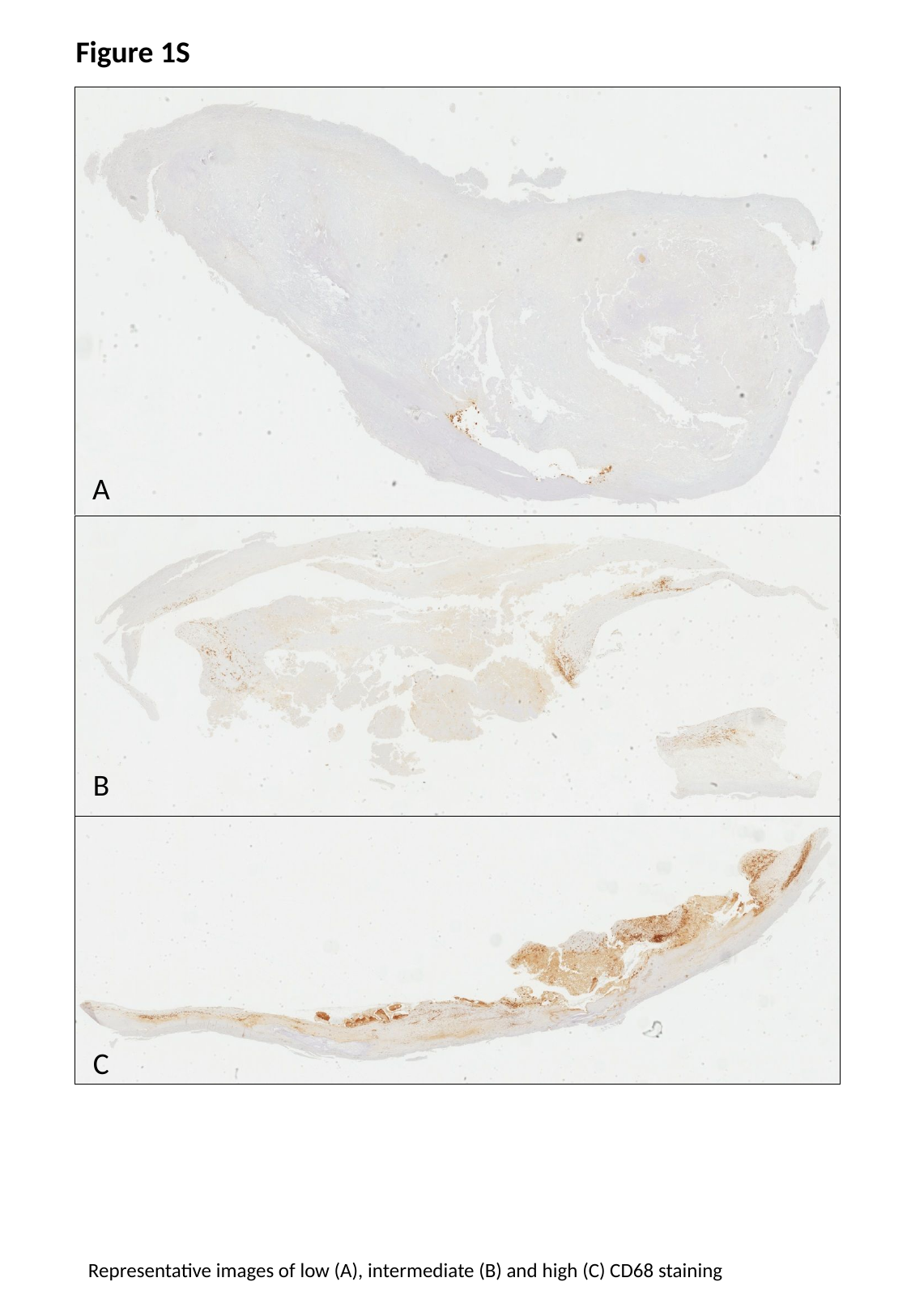

Figure 1S
A
B
C
Representative images of low (A), intermediate (B) and high (C) CD68 staining

## Slide 2
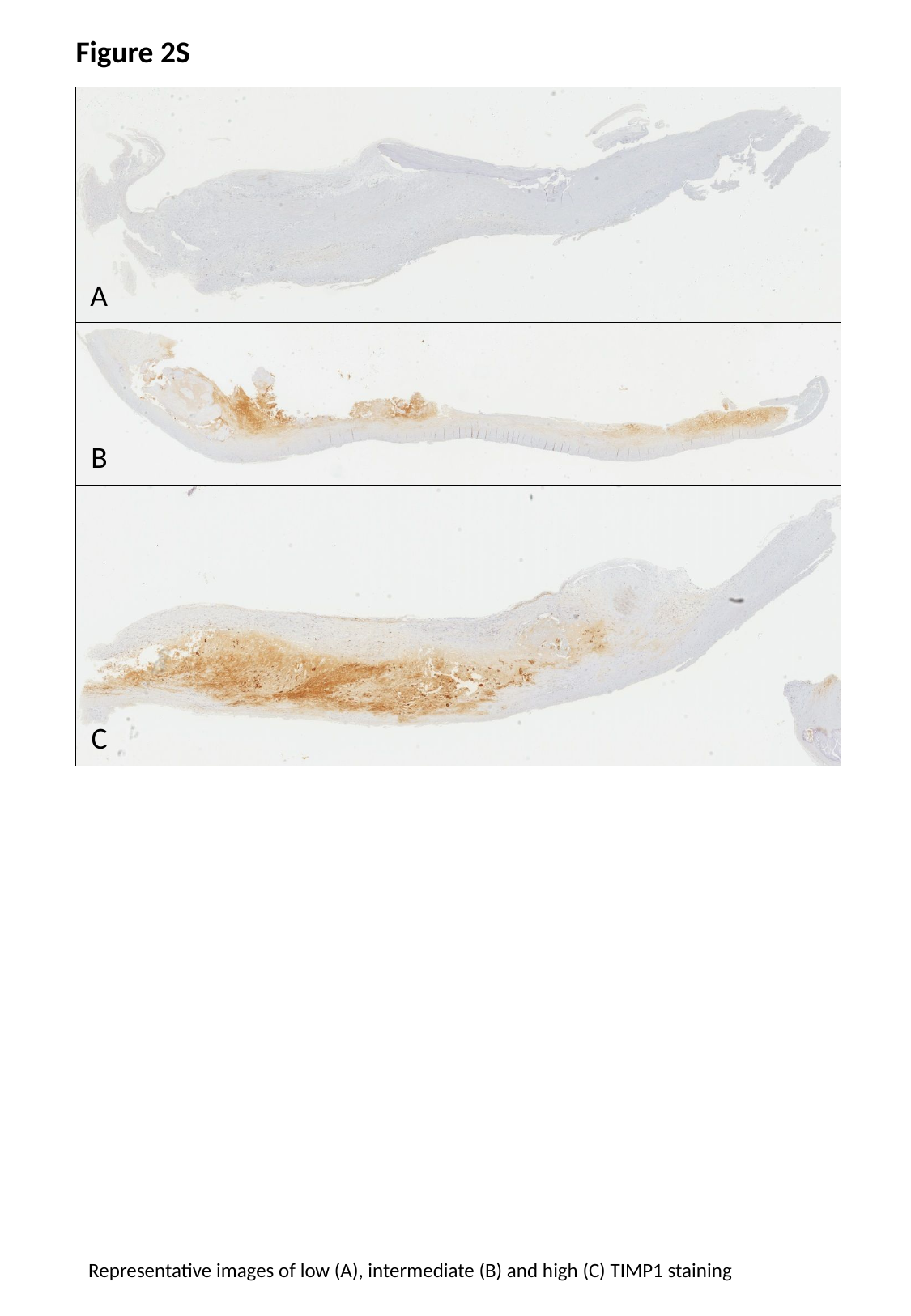

Figure 2S
A
B
C
Representative images of low (A), intermediate (B) and high (C) TIMP1 staining

## Slide 3
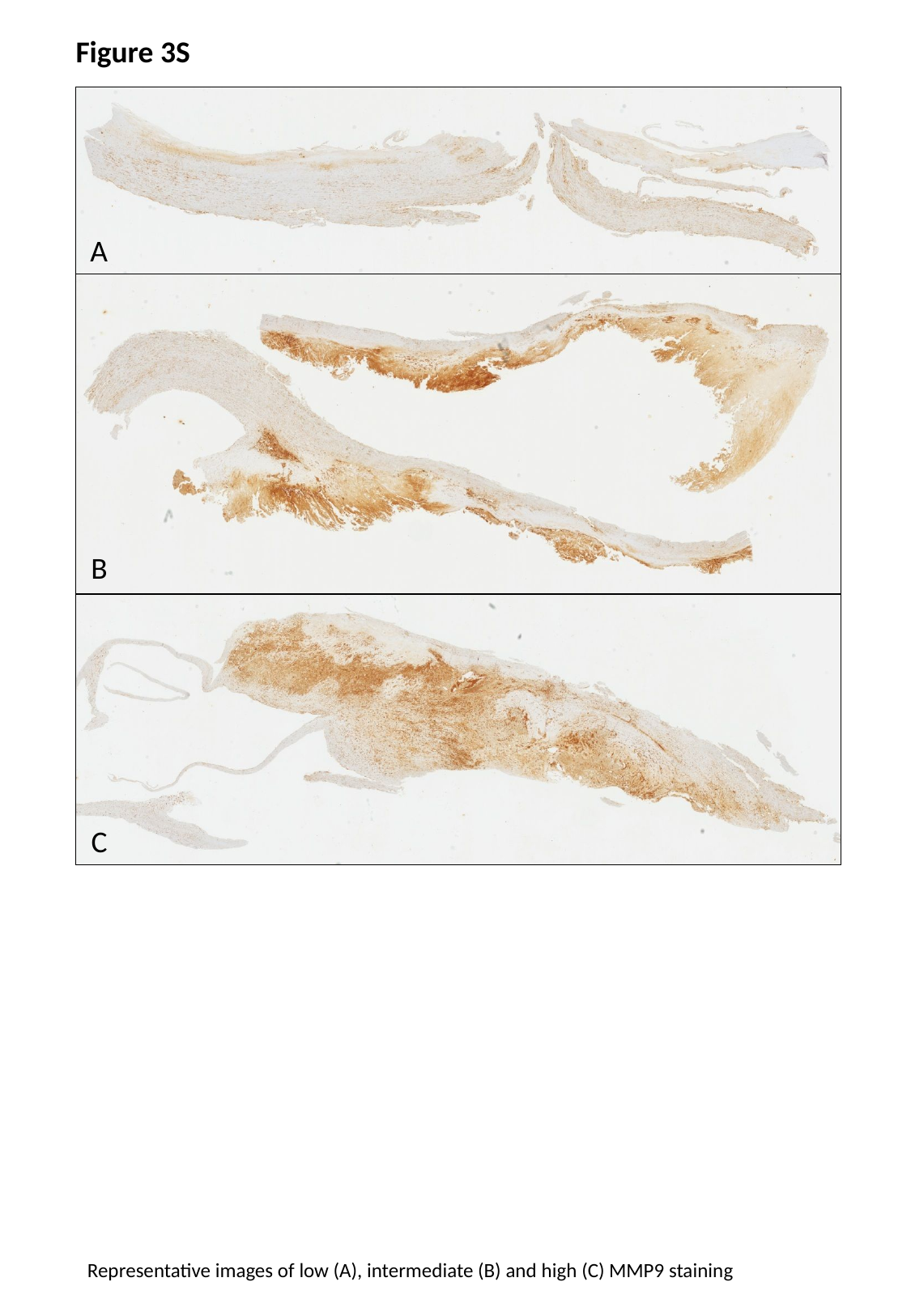

Figure 3S
A
B
C
Representative images of low (A), intermediate (B) and high (C) MMP9 staining

## Slide 4
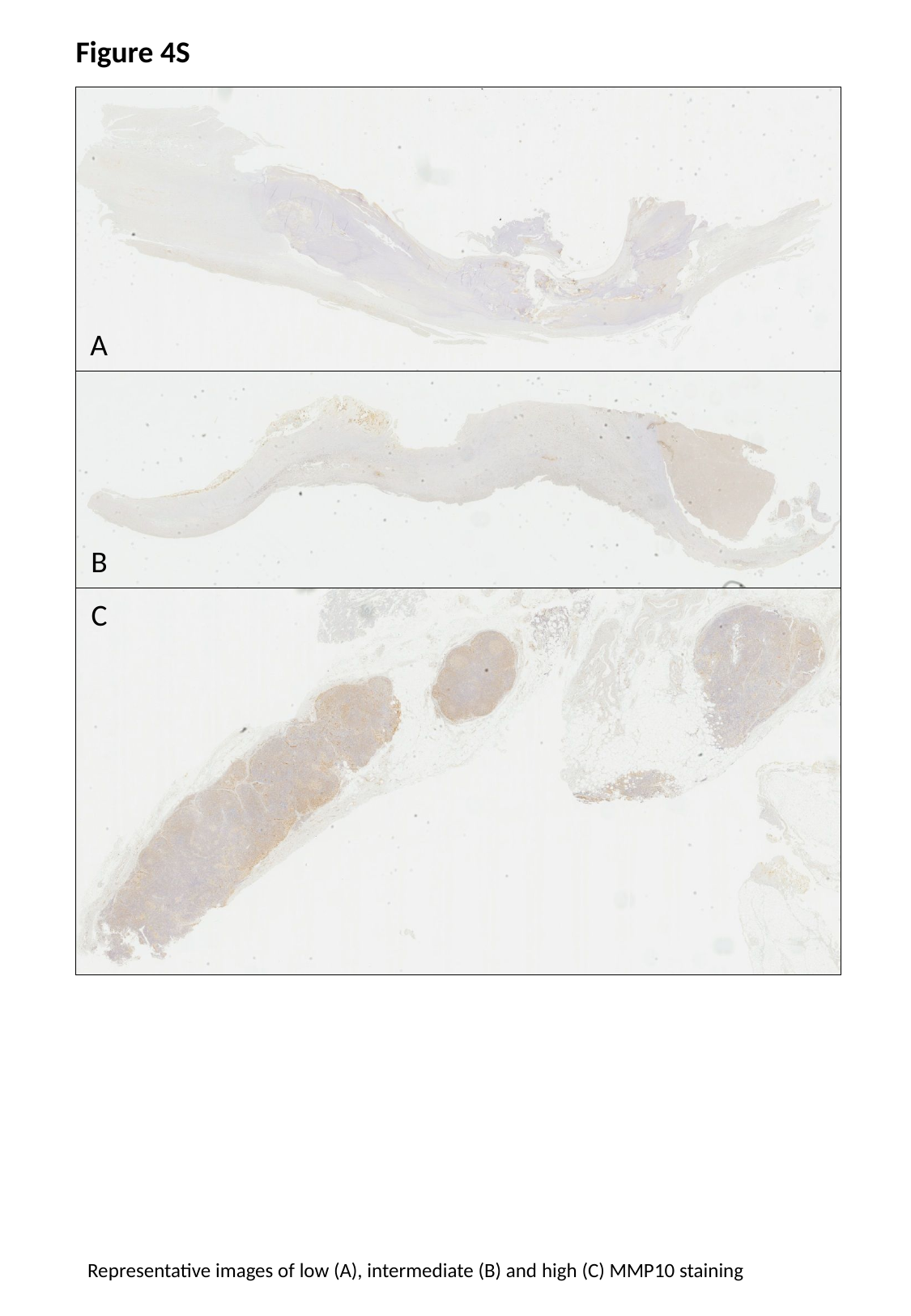

Figure 4S
A
B
C
Representative images of low (A), intermediate (B) and high (C) MMP10 staining
